# Supplementary material for: Hot spot prediction in protein-protein interactions by an ensemble system
Source: BMC Syst Biol. 2018 Dec 31;12(Suppl 9):132. doi: 10.1186/s12918-018-0665-8 (PMC6311905; doi:10.1186/s12918-018-0665-8)
Supplement: Supplementary file 2 — All relevant MATLAB code. A simple Matlab implement of our predictor. (ZIP 19,742 kb) [file 12918_2018_665_MOESM2_ESM.zip › Additional file 2/Read ASA/ASA Property/BID/2NMBA.txt]

 NetSurfP - Protein Surface Accessibility and Secondary Structure Predictions

  

|  |  |  |
| --- | --- | --- |
|  |  | NetSurfP - Protein Surface Accessibility and Secondary Structure Predictions  Technical University of Denmark |

  


---

```
# For publication of results, please cite:
# A generic method for assignment of reliability scores applied to solvent accessibility predictions.
# Bent Petersen, Thomas Nordahl Petersen, Pernille Andersen, Morten Nielsen and Claus Lundegaard
# BMC Structural Biology 2009, 9:51 doi:10.1186/1472-6807-9-51
#
# Column 1: Class assignment - B for buried or E for Exposed - Threshold: 25% exposure, but not based on RSA
# Column 2: Amino acid
# Column 3: Sequence name
# Column 4: Amino acid number
# Column 5: Relative Surface Accessibility - RSA
# Column 6: Absolute Surface Accessibility
# Column 7: Z-fit score for RSA prediction
# Column 8: Probability for Alpha-Helix
# Column 9: Probability for Beta-strand
# Column 10: Probability for Coil
E G  2NMBA                  1    0.916  72.105  -2.121   0.003   0.003   0.994
E S  2NMBA                  2    0.611  71.586  -1.031   0.005   0.015   0.979
E P  2NMBA                  3    0.572  81.110  -3.008   0.005   0.015   0.979
E G  2NMBA                  4    0.606  47.724  -1.163   0.018   0.019   0.964
E I  2NMBA                  5    0.344  63.658  -1.000   0.018   0.019   0.964
E P  2NMBA                  6    0.485  68.878  -0.693   0.018   0.019   0.964
E D  2NMBA                  7    0.588  84.731  -1.690   0.018   0.019   0.964
E R  2NMBA                  8    0.554 126.935  -0.047   0.018   0.019   0.964
E V  2NMBA                  9    0.425  65.323  -0.566   0.018   0.019   0.964
E P  2NMBA                 10    0.431  61.116  -1.002   0.018   0.019   0.964
E E  2NMBA                 11    0.717 125.207  -1.959   0.018   0.019   0.964
E S  2NMBA                 12    0.566  66.347  -1.532   0.018   0.019   0.964
E S  2NMBA                 13    0.571  66.968  -1.357   0.018   0.019   0.964
E K  2NMBA                 14    0.566 116.426   0.853   0.018   0.019   0.964
E P  2NMBA                 15    0.505  71.631  -0.528   0.018   0.019   0.964
E H  2NMBA                 16    0.596 108.412  -0.343   0.018   0.019   0.964
E Q  2NMBA                 17    0.645 115.108   0.340   0.058   0.017   0.925
B W  2NMBA                 18    0.258  62.049  -1.053   0.018   0.047   0.935
E Q  2NMBA                 19    0.498  88.996  -0.815   0.018   0.047   0.935
E A  2NMBA                 20    0.279  30.757  -0.531   0.018   0.019   0.964
E D  2NMBA                 21    0.526  75.797   0.755   0.053   0.005   0.942
E E  2NMBA                 22    0.576 100.697   0.169   0.502   0.002   0.495
E E  2NMBA                 23    0.584 102.095  -0.239   0.622   0.015   0.363
E A  2NMBA                 24    0.372  41.016   0.822   0.522   0.016   0.462
B V  2NMBA                 25    0.106  16.338  -0.585   0.561   0.047   0.393
E R  2NMBA                 26    0.413  94.577   0.200   0.354   0.048   0.598
E S  2NMBA                 27    0.646  75.735  -0.372   0.113   0.043   0.844
E A  2NMBA                 28    0.636  70.054  -1.276   0.018   0.047   0.935
E T  2NMBA                 29    0.426  59.045  -0.879   0.005   0.262   0.733
B C  2NMBA                 30    0.133  18.701  -0.033   0.003   0.718   0.279
E S  2NMBA                 31    0.318  37.293   0.598   0.001   0.900   0.099
B F  2NMBA                 32    0.070  14.009   1.000   0.001   0.959   0.040
E S  2NMBA                 33    0.340  39.801   1.403   0.001   0.900   0.099
B V  2NMBA                 34    0.023   3.581   0.919   0.001   0.959   0.040
E K  2NMBA                 35    0.315  64.775   1.527   0.001   0.959   0.040
B Y  2NMBA                 36    0.125  26.670   0.362   0.001   0.959   0.040
B L  2NMBA                 37    0.116  21.221   0.581   0.021   0.756   0.223
B G  2NMBA                 38    0.110   8.649  -1.817   0.022   0.552   0.426
B C  2NMBA                 39    0.207  29.091  -0.647   0.022   0.552   0.426
B V  2NMBA                 40    0.167  25.653   1.031   0.021   0.756   0.223
E E  2NMBA                 41    0.366  63.888   0.233   0.021   0.756   0.223
B V  2NMBA                 42    0.048   7.454   0.739   0.022   0.359   0.619
E F  2NMBA                 43    0.513 102.899  -0.278   0.019   0.141   0.840
E E  2NMBA                 44    0.553  96.696  -0.044   0.018   0.088   0.893
B S  2NMBA                 45    0.293  34.386  -1.520   0.018   0.047   0.935
E R  2NMBA                 46    0.535 122.446   0.020   0.018   0.019   0.964
B G  2NMBA                 47    0.265  20.855  -0.563   0.181   0.016   0.803
E M  2NMBA                 48    0.422  84.402   0.533   0.923   0.002   0.076
E Q  2NMBA                 49    0.575 102.784   1.419   0.970   0.001   0.030
B V  2NMBA                 50    0.099  15.186   1.095   0.970   0.001   0.030
B C  2NMBA                 51    0.015   2.148   1.507   0.988   0.000   0.012
E E  2NMBA                 52    0.363  63.399   1.881   0.988   0.000   0.012
E E  2NMBA                 53    0.492  85.900   2.141   0.988   0.000   0.012
B A  2NMBA                 54    0.024   2.634   0.815   0.988   0.000   0.012
B L  2NMBA                 55    0.036   6.610   0.159   0.970   0.001   0.030
E K  2NMBA                 56    0.485  99.867   1.974   0.988   0.000   0.012
E V  2NMBA                 57    0.378  58.114   2.490   0.988   0.000   0.012
B L  2NMBA                 58    0.031   5.768   0.312   0.970   0.001   0.030
E R  2NMBA                 59    0.311  71.150   1.086   0.970   0.001   0.030
E Q  2NMBA                 60    0.577 102.999   0.093   0.923   0.002   0.076
E S  2NMBA                 61    0.577  67.660   0.369   0.600   0.003   0.397
E R  2NMBA                 62    0.484 110.836  -0.685   0.176   0.004   0.820
E R  2NMBA                 63    0.616 140.950   0.408   0.018   0.019   0.964
E R  2NMBA                 64    0.617 141.362   0.534   0.018   0.019   0.964
E P  2NMBA                 65    0.287  40.683   0.048   0.005   0.045   0.951
E V  2NMBA                 66    0.394  60.542   0.204   0.004   0.138   0.858
E R  2NMBA                 67    0.461 105.638   1.523   0.004   0.616   0.381
B G  2NMBA                 68    0.027   2.125   0.390   0.001   0.900   0.099
E L  2NMBA                 69    0.231  42.259   1.320   0.001   0.959   0.040
B L  2NMBA                 70    0.023   4.193   0.770   0.000   0.983   0.017
E H  2NMBA                 71    0.290  52.824   1.459   0.001   0.959   0.040
B V  2NMBA                 72    0.027   4.104   0.933   0.001   0.959   0.040
B S  2NMBA                 73    0.110  12.845   0.494   0.004   0.616   0.381
E G  2NMBA                 74    0.383  30.174  -0.219   0.005   0.045   0.951
E D  2NMBA                 75    0.520  75.004   0.416   0.005   0.015   0.979
B G  2NMBA                 76    0.112   8.791   0.036   0.004   0.616   0.381
B L  2NMBA                 77    0.028   5.145   0.453   0.001   0.900   0.099
E R  2NMBA                 78    0.264  60.410   1.727   0.000   0.983   0.017
B V  2NMBA                 79    0.038   5.779   0.137   0.000   0.983   0.017
B V  2NMBA                 80    0.141  21.733   0.828   0.001   0.959   0.040
B D  2NMBA                 81    0.184  26.529  -0.213   0.003   0.718   0.279
E D  2NMBA                 82    0.483  69.543  -0.233   0.020   0.205   0.775
E E  2NMBA                 83    0.709 123.792   0.084   0.018   0.019   0.964
E T  2NMBA                 84    0.524  72.707  -0.405   0.018   0.019   0.964
E K  2NMBA                 85    0.633 130.208   0.316   0.018   0.019   0.964
E G  2NMBA                 86    0.466  36.674   0.195   0.020   0.205   0.775
E L  2NMBA                 87    0.275  50.298   1.301   0.022   0.552   0.426
B I  2NMBA                 88    0.204  37.685  -0.095   0.023   0.655   0.322
B V  2NMBA                 89    0.258  39.685   0.162   0.022   0.552   0.426
E D  2NMBA                 90    0.478  68.865  -0.632   0.022   0.552   0.426
B Q  2NMBA                 91    0.137  24.540   1.159   0.022   0.552   0.426
E T  2NMBA                 92    0.359  49.752   0.772   0.004   0.197   0.799
B I  2NMBA                 93    0.059  10.915   0.427   0.005   0.262   0.733
E E  2NMBA                 94    0.325  56.708   0.551   0.004   0.197   0.799
B K  2NMBA                 95    0.255  52.474   0.847   0.004   0.420   0.576
B V  2NMBA                 96    0.017   2.613   1.428   0.001   0.900   0.099
B S  2NMBA                 97    0.045   5.215  -0.024   0.001   0.900   0.099
B F  2NMBA                 98    0.043   8.630   0.505   0.001   0.959   0.040
B C  2NMBA                 99    0.021   2.892   0.694   0.011   0.918   0.071
B A  2NMBA                100    0.032   3.548  -1.330   0.003   0.718   0.279
B P  2NMBA                101    0.230  32.580  -0.140   0.004   0.420   0.576
B D  2NMBA                102    0.200  28.834   0.327   0.004   0.085   0.910
E R  2NMBA                103    0.527 120.775  -0.776   0.005   0.015   0.979
E N  2NMBA                104    0.679  99.464   0.042   0.018   0.019   0.964
E H  2NMBA                105    0.396  72.014   0.083   0.018   0.047   0.935
E E  2NMBA                106    0.363  63.469   0.837   0.004   0.197   0.799
E R  2NMBA                107    0.320  73.234   1.064   0.003   0.718   0.279
B G  2NMBA                108    0.036   2.857   0.643   0.001   0.900   0.099
B F  2NMBA                109    0.021   4.295   1.152   0.001   0.959   0.040
B S  2NMBA                110    0.016   1.875   0.584   0.001   0.959   0.040
B Y  2NMBA                111    0.050  10.642   0.907   0.001   0.959   0.040
B I  2NMBA                112    0.022   3.996   0.893   0.001   0.959   0.040
B C  2NMBA                113    0.039   5.476  -0.873   0.002   0.816   0.182
E R  2NMBA                114    0.284  65.013   0.234   0.003   0.718   0.279
E D  2NMBA                115    0.257  37.019   0.601   0.005   0.262   0.733
E G  2NMBA                116    0.646  50.832   0.239   0.005   0.015   0.979
E T  2NMBA                117    0.742 102.902  -1.124   0.005   0.015   0.979
E T  2NMBA                118    0.612  84.843  -0.340   0.005   0.015   0.979
E R  2NMBA                119    0.533 121.943  -0.283   0.005   0.045   0.951
E R  2NMBA                120    0.463 105.912   1.581   0.004   0.420   0.576
B W  2NMBA                121    0.092  22.102   0.601   0.002   0.816   0.182
B M  2NMBA                122    0.123  24.652   1.188   0.001   0.959   0.040
B C  2NMBA                123    0.018   2.583   1.111   0.001   0.959   0.040
B H  2NMBA                124    0.039   7.112   0.692   0.001   0.959   0.040
B G  2NMBA                125    0.023   1.794   0.970   0.001   0.959   0.040
B F  2NMBA                126    0.031   6.161   0.459   0.001   0.959   0.040
B L  2NMBA                127    0.200  36.638   1.343   0.001   0.959   0.040
B A  2NMBA                128    0.025   2.777   0.512   0.003   0.606   0.390
E C  2NMBA                129    0.415  58.266   0.894   0.004   0.085   0.910
E K  2NMBA                130    0.603 124.078   0.641   0.257   0.016   0.727
E D  2NMBA                131    0.646  93.045   0.575   0.694   0.003   0.303
E S  2NMBA                132    0.281  32.910   0.608   0.694   0.003   0.303
B G  2NMBA                133    0.032   2.542  -1.030   0.923   0.002   0.076
E E  2NMBA                134    0.401  70.002   1.279   0.970   0.001   0.030
E R  2NMBA                135    0.416  95.195   2.174   0.970   0.001   0.030
B L  2NMBA                136    0.023   4.120   1.426   0.988   0.000   0.012
B S  2NMBA                137    0.045   5.251   0.423   0.988   0.000   0.012
E H  2NMBA                138    0.378  68.776   1.813   0.988   0.000   0.012
B A  2NMBA                139    0.060   6.601   0.885   0.988   0.000   0.012
B V  2NMBA                140    0.023   3.581   1.301   0.988   0.000   0.012
B G  2NMBA                141    0.152  11.970   0.338   0.970   0.001   0.030
E C  2NMBA                142    0.347  48.691   1.209   0.970   0.001   0.030
B A  2NMBA                143    0.039   4.320   0.118   0.970   0.001   0.030
B F  2NMBA                144    0.046   9.232   0.595   0.970   0.001   0.030
E A  2NMBA                145    0.377  41.578   1.970   0.970   0.001   0.030
E V  2NMBA                146    0.253  38.917   1.224   0.970   0.001   0.030
B C  2NMBA                147    0.074  10.376   0.561   0.970   0.001   0.030
B L  2NMBA                148    0.132  24.133   0.705   0.970   0.001   0.030
E E  2NMBA                149    0.532  92.975   1.317   0.970   0.001   0.030
E R  2NMBA                150    0.460 105.386   1.583   0.970   0.001   0.030
B K  2NMBA                151    0.170  34.969   0.774   0.923   0.002   0.076
E Q  2NMBA                152    0.305  54.509   1.014   0.782   0.003   0.216
E R  2NMBA                153    0.701 160.575   1.234   0.600   0.003   0.397
E R  2NMBA                154    0.533 122.149   1.248   0.502   0.002   0.495
E T  2NMBA                155    0.588  81.597   0.685   0.406   0.004   0.590
E R  2NMBA                156    0.618 141.614   0.631   0.406   0.004   0.590
E A  2NMBA                157    0.624  68.743  -0.291   0.406   0.004   0.590
E A  2NMBA                158    0.582  64.114  -0.920   0.246   0.004   0.750
E A  2NMBA                159    0.730  80.490  -0.739   0.109   0.005   0.886
E S  2NMBA                160    0.856 100.311  -0.397   0.003   0.003   0.994

Explain the output.  Go back.
```

---
